# “Efficacy and safety of bimekizumab in moderate-to-severe hidradenitis suppurativa: A systematic review and meta-analysis of RCTs”

**DOI:** 10.1097/MD.0000000000047691

**Published:** 2026-02-13

**Authors:** Ahmad Assiri, Muhammad Almahdi, Osama Mobarki, Rimas Sumayli, Nawaf Alharbi, Wesam Alharbi, Suhayb Alhazmi, Abdulaziz Alghamdi, Mohammed Alturkistani

**Affiliations:** a Department of Dermatology, Faculty of Medicine, Jazan University, Jazan, Saudi Arabia; b Faculty of Medicine, Jazan University, Jazan, Saudi Arabia; c Faculty of Medicine, Umm Al-Qura University, Makkah, Saudi Arabia.

**Keywords:** bimekizumab, biologics, efficacy, hidradenitis suppurativa, safety

## Abstract

**Background:**

Hidradenitis suppurativa (HS) is a chronic inflammatory dermatosis with limited treatment options. This systematic review and meta-analysis aimed to evaluate the evidence regarding the use of bimekizumab for treating moderate-to-severe HS, focusing on its clinical efficacy, safety, and outcomes across different clinical parameters.

**Methods:**

We followed the “preferred reporting items for systematic reviews and meta-analyses” (PRISMA) guidelines and performed a systematic search for trials that compared bimekizumab with a placebo in patients with moderate-to-severe HS. A comprehensive search of PubMed, Web of Science, Cochrane CENTRAL, and Embase was conducted up to April 2025. The primary outcome of efficacy was the percentage of patients who reached “HS Clinical Response 50” (HiSCR50), with secondary outcomes including HiSCR75, reduction in skin pain, and safety (evaluated by serious adverse events). Two reviewers independently assessed the risk of bias using the Cochrane risk-of-bias 2 tool. A fixed-effects model was used for meta-analysis. The study protocol was preregistered in PROSPERO (CRD420251025763).

**Results:**

Three RCTs, encompassing 1218 participants, were included. Bimekizumab made it substantially more probable that the HiSCR50 would be reached (RR: 1.64; 95% CI [1.37–1.97]; *P* <.00001) than the placebo. Patients who received bimekizumab were also substantially more likely to achieve higher response levels, HiSCR75 (RR: 1.98; 95% CI [1.52–2.59]; *P* <.00001) and a considerable reduction in skin pain (RR: 2.29; 95% CI [1.54–3.42]; *P* <.0001). For all measures of efficacy, both the every-two-week and every-four-week dosage schedules were better than the placebo. The occurrence of serious adverse events did not differ significantly between the bimekizumab and placebo groups (RR: 2.34; 95% CI [0.80–6.79]; *P* = .12).

**Conclusion:**

The evidence, although derived from a limited number of trials, demonstrates the superiority of bimekizumab over placebo. For patients with moderate-to-severe HS, bimekizumab showed significantly improved outcomes compared to placebo in terms of enhancing clinical response and lessening skin pain. Although no statistically significant increase in serious adverse events was observed, a potential risk cannot be definitively ruled out given the numerical imbalance.

## 1. Introduction

Chronic inflammatory dermatosis, known as hidradenitis suppurativa (HS), primarily “affects areas with a high density of apocrine glands and is characterized by recurrent painful nodules, abscesses, and sinus tracts.”^[1,2]^ It begins as a subcutaneous nodule and frequently progresses to painful fistulas and abscesses with purulent discharge.^[3]^ This long-term illness has a substantial “negative influence on quality of life and causes psychological and physical suffering.”^[4–8]^ Due to the burden of symptoms, recurrent HS can cause contractures, decreased mobility, and chronic pain.^[9]^ Amyloidosis, spondyloarthropathy, and anemia are examples of long-term complications.^[9,10]^ Furthermore, squamous cell carcinoma may develop as a complication.^[11]^ Anxiety, depression, social isolation, sexual dysfunction, and possibly suicide are examples of psychological complications.^[9,12,13]^

It is not yet fully known how HS develops.^[3]^ However, it is linked to the “overexpression of cytokines, including interleukin (IL)-1, IL-17, IL-23, tumor necrosis factor-alpha (TNF-α), and other inflammatory mediators.”^[14]^ Furthermore, “keratinocytes and immune cells, such as T Helper 1 and 17 lymphocytes, contribute to its development.”^[15]^

HS can be treated with antibiotics, retinoids, intralesional corticosteroids, biologics, and surgery.^[3,16,17]^ Despite the possibility of disfigurement, extensive surgical intervention may be required in some cases.^[18]^ Biologic agents may improve disease activity metrics and reduce HS related pain, according to clinical evidence.^[19]^

Bimekizumab, a novel treatment for HS, selectively inhibits IL-17F and IL-17A, which have been implicated in the inflammatory processes of HS. Recent phase III trials, the “BE HEARD I and BE HEARD II” have established the safety and effectiveness of bimekizumab in the management of HS.^[20]^ These conclusions are supported by data from real-world effectiveness studies, which show improvements in patient-reported outcomes and disease activity scores.^[21]^ This systematic review and meta-analysis examined the evidence on using bimekizumab for HS, with attention to its clinical effectiveness, safety profile, and outcomes across various clinical parameters.

## 2. Methods

### 2.1. Registration

We adhered to the PRISMA guidelines for study selection, data extraction, and reporting to maintain a transparent and systematic process.^[22]^ Additionally, we followed the “Cochrane Handbook for Systematic Reviews” to adhere to methodological standards.^[23]^ To further enhance transparency and reduce bias, we preregistered the study protocol in the PROSPERO database under the identifier CRD420251025763.^[24]^

### 2.2. Search strategy

We developed a comprehensive search strategy using text words and “Medical Subject Headings (MeSH terms)” across all relevant fields. Search terms included: (“HS” OR “Acne Inversa” OR “Verneuil Disease”) AND (“bimekizumab” OR “UCB4940” OR “IL-17 inhibitor” OR “interleukin-17 inhibitor”). The search included all available literature from inception to April 2025, with no restrictions on the publication date.

### 2.3. Selection of studies

Rayyan Software was used for the initial screening of articles based on title and abstract.^[25]^ We included studies if they met the following: patients with moderate-to-severe HS treated with bimekizumab, only RCTs were considered eligible, studies had to report relevant clinical outcomes addressing the research questions, and only English-language publications were included. Studies were excluded if they: did not evaluate bimekizumab for moderate-to-severe HS; were editorials, letters, commentaries, case reports/series, reviews, or observational studies; reported irrelevant outcomes that were not aligned with the study objectives; were published in non-English languages; or exhibited a high risk of bias or methodological flaws.

### 2.4. Data extraction

Two team members performed data extraction using a standardized form to ensure accuracy and consistency. Inconsistencies were addressed through discussions with input from a senior author when necessary. From each included study, the following variables were retrieved: trial registration number, study design and phase of trial, dosing regimen, method of delivery, number of patients in each arm, primary and secondary efficacy outcome measures, safety outcome measures, follow-up duration, event counts, and total sample size. The extracted data were cross-verified against the original publications and trial registries to ensure fidelity.

### 2.5. Bias and quality assessment

Two team members independently assessed the risk of bias using the “Cochrane risk-of-bias 2” tool. This tool enabled the analysis of multiple aspects, such as “randomization, concealment of allocation, blinding of participants and staff, outcome evaluators’ blinding, management of missing data, and selective reporting.”^[26]^ Discrepancies were resolved by the senior author.

### 2.6. Data synthesis and statistical analysis

Statistical analyses were done using “Review Manager (RevMan) version 5.4.1” software. The main outcome used to assess efficacy was the attainment of “HS Clinical Response 50 (HiSCR50)” at the study endpoint, which was analyzed as dichotomous data. The reported serious adverse events (SAEs) were evaluated in a similar manner. Risk ratios (RRs) with 95% confidence intervals (CIs) were calculated to compare bimekizumab (both every 2 weeks, Q2W; and every 4 weeks, Q4W regimens) with placebo. The RRs with 95% CIs were computed for SAEs. The I^2^ statistic and Chi^[2]^ test (*P* <.10 considered significant) were used to assess heterogeneity. For analyses demonstrating low heterogeneity, a fixed-effects model was selected to pool the data (I^2^ <50%). Subgroup analyses were performed according to dosing regimen (Q2W vs Q4W). Results were deemed significant if the 95% CI for RR excluded 1.0 or *P* <.05. Forest plots were generated to visually summarize the effect estimates and heterogeneity. Funnel plots were not drawn since there were <10 studies included.

### 2.7. Ethical considerations

Ethical approval was not required for this study as it involves the retrieval and synthesis of data from previously published studies. No interaction with human participants was involved; therefore, informed consent was not required.

## 3. Results

### 3.1. Search results

**Figure 1.**
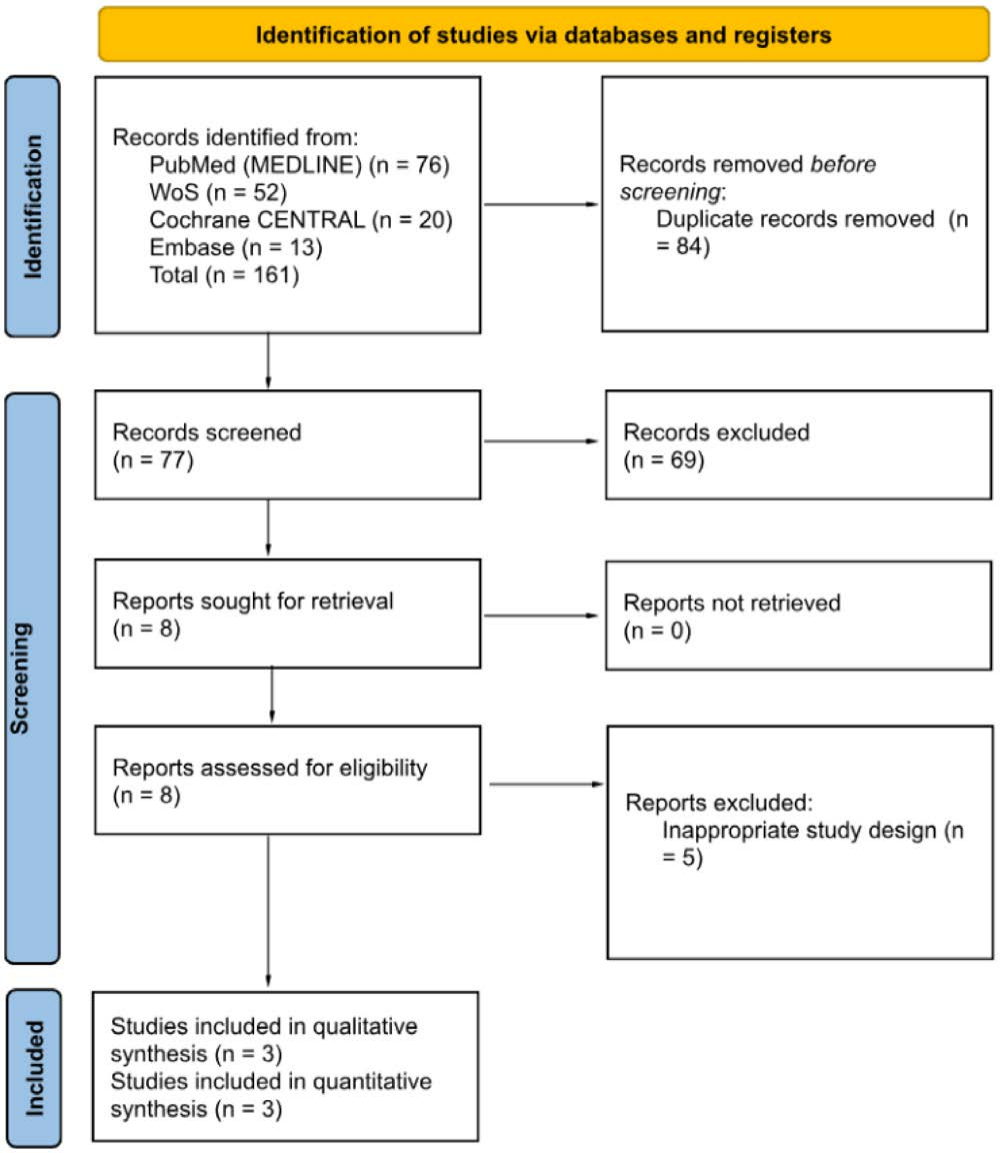
Flowchart of the reviewed studies according to PRISMA. PRISMA = preferred reporting items for systematic reviews and meta-analyses.

The systematic database search retrieved a total of 161 records: 76 from PubMed, 52 from Web of Science, 20 from Cochrane CENTRAL, and 13 from Embase. After removing 84 duplicate records, 77 studies underwent titles and abstracts screening. Sixty-nine studies were excluded during the screening phase because they did not satisfy the predefined eligibility criteria. Eight retrieved studies underwent full-text eligibility assessments. At this stage, 5 studies were excluded due to inappropriate study designs. Ultimately, 3 RCTs published between 2021 and 2024 were included because they satisfied all inclusion criteria.^[20,27]^ The process is summarized in the flowchart (Fig. 1).

### 3.2. Characteristics of the included studies

This systematic review included 3 randomized, double-blind, placebo-controlled trials that evaluated how effective and safe bimekizumab is for patients with HS. These were 1 phase II trial by Glatt et al (2021) (NCT03248531) and 2 phase III trials by Kimball et al (2024), titled “BE HEARD I (NCT04242446)” and “BE HEARD II (NCT04242498).”^[20,27]^ In the Glatt et al (2021) trial, 46 patients in the bimekizumab group received “subcutaneous injections of 640 mg at week 0, followed by 320 mg every 2 weeks, while 22 patients were assigned to the placebo group; the treatment period lasted 12 weeks, followed by a 20-week safety follow-up.”^[27]^ The BE HEARD trials were larger studies, with BE HEARD I randomizing 505 patients (289 to bimekizumab 320 mg Q2W, 144 to bimekizumab 320 mg Q4W, and 72 to placebo) and BE HEARD II randomizing 509 patients (291 to Q2W, 144 to Q4W, and 74 to placebo).^[20]^ Both BE HEARD trials featured an initial treatment period for 16 weeks and were followed by a maintenance period for 32 weeks.^[20]^

All trials assessed efficacy primarily through the proportion of patients who achieved HiSCR50. Secondary efficacy outcomes in the Glatt et al^[27]^ trial included higher response levels (HiSCR75, HiSCR90), change in “Patient Global Assessment (PtGA)” of skin pain from baseline, and the proportion of patients who made it to a “Dermatology Life Quality Index (DLQI)” score of zero or 1 (indicating remission). The BE HEARD trials extended these outcomes to include HiSCR100, the absolute change in DLQI scores, and skin pain as measured by the **“**HS Symptom Daily Diary**”** (HSSDD). Safety was evaluated by adverse events. Table 1 summarizes the characteristics of the included studies.

**Table 1:** Baseline characteristics of the included studies.

Study		Glatt et al^[27]^	Kimball et al^[20]^ (BE HEARD I)	Kimball et al^[20]^ (BE HEARD II)
Trial registration identifier		NCT03248531	NCT04242446	NCT04242498
Design		Randomized, double-blind, placebo-controlled, phase II trial	Randomized, double-blind, placebo-controlled, multicentre phase III trial	Randomized, double-blind, placebo-controlled, multicentre phase III trial
Bimekizumab	Patient Numbers	46	Q2W: 289, Q4W: 144	Q2W: 291, Q4W: 144
	Method of delivery	Subcutaneous injections of bimekizumab. The regimen was 640 mg at week 0, followed by 320 mg Q2W	Subcutaneous injection of bimekizumab 320 mg	Subcutaneous injection of bimekizumab 320 mg
Placebo	Patient Numbers	22	72	74
	Method of delivery	Subcutaneous injections of placebo weekly from week 4, with initial doses at baseline and week 2 to maintain blinding	Subcutaneous injection of placebo Q2W	Subcutaneous injection of placebo Q2W
Outcomes of Efficacy		- Proportion of patients achieving clinical response (defined as HiSCR50) - HiSCR75 - HiSCR90 - Change in PtGA of skin pain from baseline - Proportion of patients achieving DLQI 0/1 - Improvements in IHS4	- Proportion of patients achieving clinical response (defined as HiSCR50) - HiSCR75 - HiSCR90 - HiSCR100 - Absolute change from baseline in DLQI score - Absolute change from baseline in skin pain score (assessed by HSSDD)	- Proportion of patients achieving clinical response (defined as HiSCR50) - HiSCR75 - HiSCR90 - HiSCR100 - Absolute change from baseline in DLQI score - Absolute change from baseline in skin pain score (assessed by HSSDD)
Outcomes of safety		Occurrence of AEs during treatment	Occurrence of AEs during treatment	Occurrence of AEs during treatment
Follow-up, weeks		12-wk treatment, and 20-wk safety follow-up period after the final treatment dose	- Initial treatment period: wk 0–16 (16 wk). - Maintenance Treatment Period: wk 16–48 (32 wk)	- Initial treatment period: wk 0–16 (16 wk). - Maintenance treatment period: wk 16–48 (32 wk)

AEs = adverse events, DLQI = dermatology life quality index (0/1, indicates remission), HiSCR = hidradenitis suppurativa clinical response, HSSDD = hidradenitis suppurativa symptom daily diary, IHS4 = international hidradenitis suppurativa severity score, PtGA = patient global assessment, Q2W = every 2 wk, Q4W = every 4 wk.

### 3.3. Efficacy outcomes

#### 3.3.1. *Achievement of clinical response (HiSCR50*)

**Figure 2.**
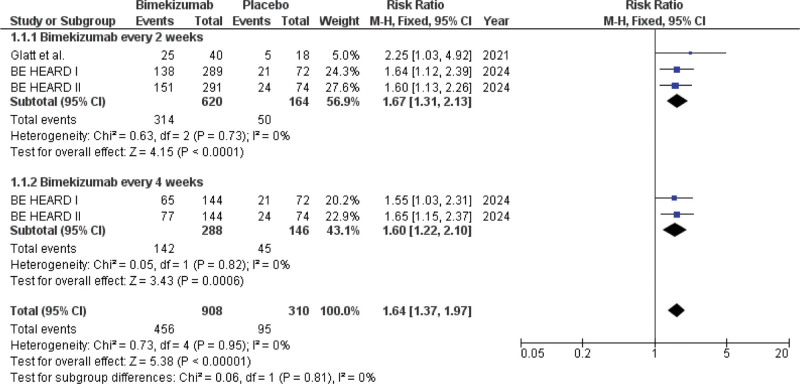
Forest plot of patients who achieved HiSCR50.

Three studies (reported across 5 data points) involving 1218 participants (908 receiving bimekizumab and 310 receiving placebo) were analyzed for the outcome of achieving HiSCR50. This analysis population represents the intention-to-treat (ITT) set for the phase 3 trials and the Per-Protocol set for the phase 2 trial. The overall pooled analysis demonstrated that the bimekizumab patient groups had a significantly higher chance of achieving HiSCR50 than the placebo group (RR: 1.64; 95% CI [1.37–1.97]; *P* <.00001) (Fig. 2).

Subgroup analysis based on bimekizumab dosing frequency: In bimekizumab Q2W, 3 study arms contributed data to this subgroup, involving 620 patients on bimekizumab and 164 on placebo. Bimekizumab Q2W patients had a significantly higher likelihood of achieving HiSCR50 than placebo patients (RR: 1.67; 95% CI [1.31–2.13]; *P* <.0001). In the bimekizumab Q4W, 2 study arms provided data for this subgroup, including 288 patients on bimekizumab and 146 on placebo. The Q4W dosing regimen also showed a significant benefit over the placebo (RR: 1.60; 95% CI [1.22–2.10]; *P* = .0006). The test for subgroup differences did not show any significant difference in efficacy between the bimekizumab Q2W and Q4W dosing regimens (*P* = .81).

#### 3.3.2. Achievement of HiSCR75

**Figure 3.**
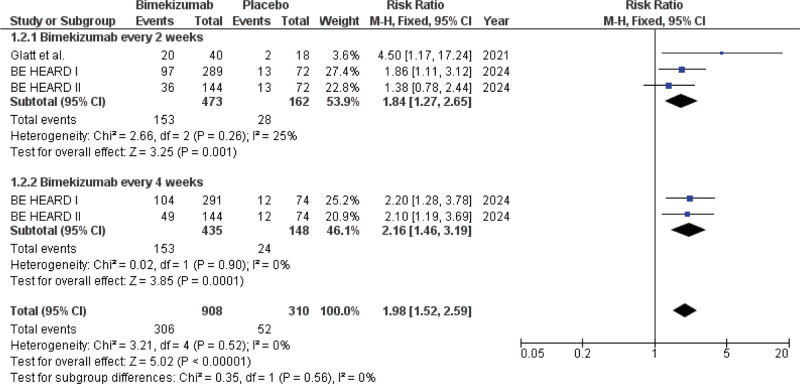
Forest plot of patients who achieved HiSCR75.

The meta-analysis of the HiSCR75 outcome included data from 3 studies, with 1218 participants (908 allocated to the bimekizumab group and 310 to the placebo group). Treatment with bimekizumab was associated with a significantly greater probability of attaining HiSCR75 compared to placebo, as shown in the pooled analysis (RR: 1.98; 95% CI [1.52–2.59]; *P* <.00001) (Fig. 3).

Subgroup analyses based on bimekizumab dosing frequency yielded the following results: In bimekizumab Q2W, data from 3 study arms, involving 473 patients receiving bimekizumab and 162 receiving placebo, were pooled. This subgroup showed a statistically significant benefit for bimekizumab (RR: 1.84; 95% CI [1.27–2.65]; *P* = .001). In the bimekizumab Q4W, data from 2 study arms, comprising 435 patients on bimekizumab and 148 on placebo, were analyzed. Patients receiving bimekizumab Q4W demonstrated a significantly higher chance of achieving HiSCR75 than those receiving placebo (RR: 2.16; 95% CI [1.46–3.19]; *P* = .0001). The efficacy of bimekizumab in achieving HiSCR75 did not differ significantly between the 2 dosing intervals, based on subgroup analysis (*P* = .56).

#### 3.3.3. Reduction in pain (HSSDD, PtGA)

**Figure 4.**
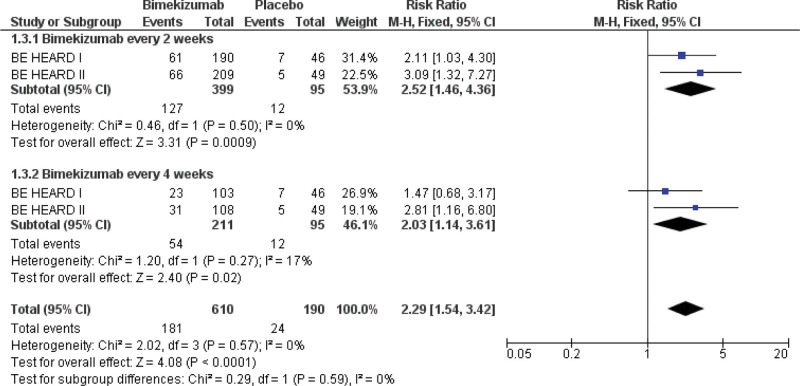
Forest plot of patients who achieved improvements in HSSDD weekly worst pain score (≥3 points). HSSDD = hidradenitis suppurativa symptom daily diary.

Data on the proportion of patients attaining at least a 3-point improvement from baseline in the HSSDD weekly worst skin pain score (among those with a baseline score ≥3) were available from 2 studies. This analysis included 800 participants (610 receiving bimekizumab and 190 receiving the placebo). Bimekizumab was associated with a significantly greater likelihood of skin pain response than placebo, as shown in the pooled analysis (RR: 2.29; 95% CI [1.54–3.42]; *P* <.0001) (Fig. 4).

Subgroup analysis based on dosing frequency showed the following: In bimekizumab Q2W, data were pooled from 2 study arms (399 bimekizumab and 95 placebo). Patients who received bimekizumab Q2W were significantly more likely to achieve ≥3-point pain improvement than those who received placebo (RR: 2.52; 95% CI [1.46–4.36]; *P* = .0009). 2) For the bimekizumab Q4W, data were combined from 2 study arms (211 bimekizumab and 95 placebo). The Q4W dosing regimen also showed a significantly increased number of patients attaining pain response when compared to the placebo (RR: 2.03; 95% CI [1.14–3.61]; *P* = .02). Skin pain response subgroup analyses showed no significant variation (test for subgroup differences: *P* = .59).

Glatt et al^[27]^ trial assessed improvements in pain using the PtGA, which is composed of 11 points for numerically rating skin pain. At week 12, 64% of patients receiving bimekizumab reached the endpoint for pain reduction (≥30% and ≥1-unit decrease), compared to 37% in the placebo group.

#### 3.3.4. DLQI

The DLQI is a 0 to 30 scale, where 0 to 1 means no effect of HS on quality of life.^[27]^ Glatt et al^[27]^ compared patients in the bimekizumab and placebo groups who achieved a DLQI 0/1 at week 12. Of the 42 patients, 27 (36%) in the bimekizumab group achieved a DLQI of 0/1, compared with none in the placebo group. In the BE HEARD I and II trials, the absolute change in DLQI was measured at week 16; both bimekizumab regimens showed a significant improvement, with lower DLQI scores than placebo at the 16-week mark.^[20]^

### 3.4. Safety

**Figure 5.**
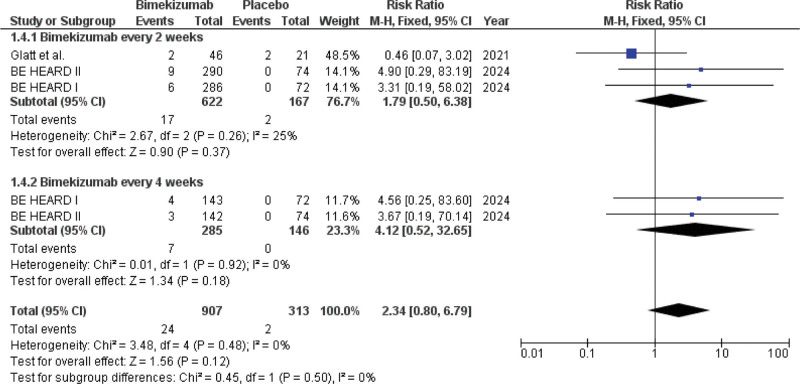
Forest plot of patients who developed SAEs. SAEs = serious adverse events.

The occurrence of SAEs was evaluated using data from 3 studies, including 1220 participants (907 receiving bimekizumab and 313 receiving placebo). The safety analysis set (n = 1220) differed slightly from the efficacy set (n = 1218). The overall incidence of SAEs was low in both groups (bimekizumab: 24/907 [2.6%]; placebo: 2/313 [0.6%]). The overall risk of SAEs did not differ significantly between patients receiving bimekizumab and those receiving placebo, based on pooled data (RR: 2.34; 95% CI [0.80–6.79]; *P* = .12) (Fig. 5).

In bimekizumab Q2W, the risk of SAEs was not significantly different in the study arms (622 patients on bimekizumab and 167 on placebo) compared to placebo (RR: 1.79; 95% CI [0.50–6.38]; *P* = .37). In bimekizumab Q4W, the risk of SAEs in the study arms (285 patients on bimekizumab and 146 on placebo) was also not significantly different than placebo (RR: 4.12; 95% CI 0.52–32.65; *P = *.18). Notably, no SAEs were reported in the placebo arms of this subgroup, contributing to a wide confidence interval. Pooled data indicated that the frequency of SAEs did not differ significantly between the 2 dosing regimens of bimekizumab (*P* = .50).

### 3.5. Quality assessment of the included studies

**Figure 6.**
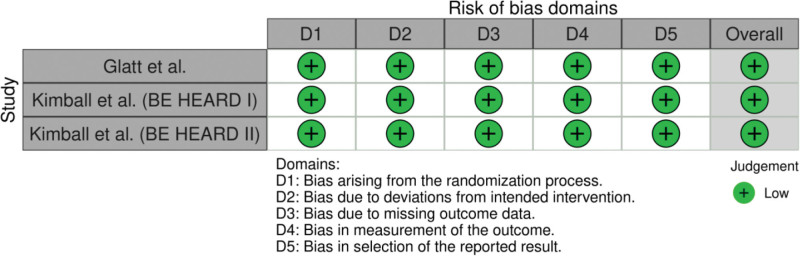
Summary of risk of bias judgements for Glatt et al and Kimball et al (BE HEARD I and II) across 5 domains.

Two reviewers independently employed the RoB2 tool to assess the risk of bias in 3 eligible studies. Figure 6, created using the robvis tool, illustrates the bias assessment across all studies.^[28]^ All 3 studies showed low risk in all domains. This means the overall risk of bias was low, pointing to strong methodological quality and minimal chance that bias affected the results.

## 4. Discussion

This systematic review and meta-analysis examined the current evidence on the use of bimekizumab compared with a placebo for HS. Data were derived from 3 RCTs.^[20,27]^ The results showed that bimekizumab significantly improved skin pain and disease activity compared to placebo.

Patients who were administered bimekizumab showed a significantly higher chance of achieving the primary efficacy outcome (HiSCR50) at the primary endpoint (weeks 12 or 16) than those who received placebo (RR: 1.64; 95% CI [1.37–1.97]; *P* <.00001). This finding holds true for all the studies. Additionally, bimekizumab showed a markedly higher chance of achieving HiSCR75 (RR: 1.98; 95% CI [1.52–2.59]; *P* <.00001), indicating its superior efficacy in reaching higher response thresholds. These robust response rates highlight the potential of bimekizumab to improve clinical outcomes in patients with HS, who are often burdened by refractory disease.^[4–8]^

Significant pain is a hallmark of HS that negatively affects patients’ quality of life.^[4,9]^ According to the meta-analysis, a significantly greater percentage of patients receiving bimekizumab treatment experienced a clinically meaningful decrease in skin pain than those who were given a placebo (RR: 2.29; 95% CI [1.54–3.42]; *P* <.0001). Moreover, qualitative evaluation of the DLQI data from the included trials indicated significant improvements in the quality of life; the BE HEARD trials showed significant decreases in DLQI scores from baseline, and Glatt et al (2021) reported a notable proportion achieving DLQI 0/1.^[20,27]^

By targeting both IL-17A and IL-17F, the distinct mechanism of bimekizumab sets it apart from other biologics used or being researched for HS, like the TNF-alpha inhibitor adalimumab or the IL-17A inhibitor secukinumab.^[19,29]^ The pathophysiology of HS is linked to both IL-17F and IL-17A, and their simultaneous inhibition may provide greater therapeutic benefit.^[14,15]^ Although direct comparisons with other biologics are out of scope in this meta-analysis, the level of HiSCR achievement seen with bimekizumab seems to be robust in the context of HS treatments.

The pooled RRs for achieving HiSCR50, HiSCR75, or pain reduction during the initial treatment period did not show statistically significant differences according to subgroup analyses based on dosing frequency. For every efficacy outcome examined, the Q2W and Q4W regimens were significantly superior to placebo. Although the point estimates favored 1 regimen over the other based on the outcome (RR for HiSCR75 was slightly higher for Q4W, and RR for pain reduction was higher for Q2W), the absence of significant subgroup differences indicates that both regimens provide a significant short-term advantage over a placebo. However, these subgroup findings should be interpreted with caution. The absence of a statistically significant difference does not confirm clinical equivalence, as the current analysis may lack the statistical power to detect minor differences between the 2 dosing schedules.

The pooled analysis across the 3 trials revealed no statistically significant increase in SAE risk for bimekizumab compared to placebo during the initial treatment phase (RR: 2.34; 95% CI [0.80–6.79]; *P* = .12). While the *P*-value indicates nonsignificance, the numerical imbalance (2.6% vs 0.6%) and the risk ratio of 2.34 warrant careful consideration. The wide confidence interval includes the possibility of an increased risk, and the analysis may be underpowered to detect rare serious events due to the low absolute event rate. Therefore, while the short-term profile appears acceptable, a potential for a modestly increased risk of SAEs with bimekizumab cannot be definitively ruled out based on current data. The analysis focused only on SAEs, so physicians should still consider the recognized side effects of these biologics.

To ensure data transparency, it is important to clarify the discrepancy in participant numbers between the safety analysis set (n = 1220) and the efficacy analysis set (n = 1218). The difference arises from variations in the statistical analysis populations used in the included studies. Safety analysis (n = 1220) represents the Safety Set across all 3 trials, defined as all randomized participants who received at least 1 dose of study treatment. Efficacy analysis (n = 1218) differs slightly due to the different approaches used in the underlying studies. The phase 3 trials (Kimball et al)^[20]^ employed an ITT analysis, which includes all randomized patients, even those who withdrew before dosing. The phase 2 trial (Glatt et al)^[27]^ used a Per-Protocol approach, restricting analyses to participants with confirmed endpoint data, excluding dropouts and missing data. The combination of ITT and Per-Protocol approaches accounts for the net difference between the total efficacy and safety sets.

Strengths of this review include adherence to PRISMA and Cochrane guidelines, preregistration, inclusion of RCTs, rigorous bias risk assessment, and the employment of meta-analysis to provide pooled effect estimates with enhanced precision. The consistency of the findings across different efficacy endpoints increased confidence in the results. However, this study has some limitations that must be acknowledged. The analysis was based on only 3 RCTs (representing 5 data points for the dosing arms). Although the 2 were large phase III trials, the limited number restricts the power to detect subtle differences in subgroup analyses and precludes a formal assessment of publication bias via funnel plots. Moreover, we relied on aggregate data reported in the publications to prevent patient-level analyses.

This meta-analysis offers compelling support for bimekizumab as an effective treatment for individuals with moderate-to-severe HS. It offers significant improvements in clinical response rates (HiSCR50 and HiSCR75), substantial pain relief, and an improved quality of life. Future studies should look at some important aspects. Studies investigating the predictors of response and exploring efficacy in specific patient subgroups would further refine the clinical application. Real-world evidence-based studies are valuable for confirming the effectiveness and safety of routine clinical practice in diverse patient populations. Furthermore, post-marketing surveillance is valuable for assessing long-term safety.

Bimekizumab was more effective than placebo in improving the clinical response (HiSCR50 and HiSCR75), lowering skin pain, and improving the quality of life in patients with moderate-to-severe HS during the initial phase of treatment. Both Q2W and Q4W regimens showed notable efficacy. The short-term safety profile did not show a statistically significant increase in SAEs; however, given the low event rates and wide CIs, larger, long-term studies are required to more definitively characterize the safety profile. Bimekizumab is a promising option for treating HS.

## Author contributions

**Conceptualization:** Ahmad Assiri, Muhammad Almahdi, Osama Mobarki, Rimas Sumayli, Suhayb Alhazmi, Abdulaziz Alghamdi, Mohammed Alturkistani.

**Data curation:** Osama Mobarki, Rimas Sumayli.

**Formal analysis:** Ahmad Assiri, Muhammad Almahdi, Rimas Sumayli.

**Investigation:** Osama Mobarki, Nawaf Alharbi, Wesam Alharbi, Mohammed Alturkistani.

**Methodology:** Ahmad Assiri, Muhammad Almahdi, Suhayb Alhazmi, Abdulaziz Alghamdi.

**Software:** Muhammad Almahdi.

**Supervision:** Ahmad Assiri.

**Validation:** Ahmad Assiri.

**Writing – original draft:** Nawaf Alharbi, Wesam Alharbi.

**Writing – review & editing:** Ahmad Assiri, Muhammad Almahdi.
